# Malignant Fibrous Histiocytoma of the Scalp with Skull Invasion: A Rare and Aggressive Presentation

**DOI:** 10.7759/cureus.7801

**Published:** 2020-04-23

**Authors:** Peter Andrawes, David A Hill, Marilyn Ng, Ami Raval, Alfred Culliford

**Affiliations:** 1 Plastic, Reconstructive and Hand Surgery, Northwell Health/Donald and Barbara Zucker School of Medicine, Staten Island University Hospital, Staten Island, USA; 2 Plastic Surgery, Houston Methodist Hospital, Houston, USA; 3 Plastic and Reconstructive Surgery, Houston Methodist Hospital, Houston, USA; 4 Neurological Surgery, Northwell Health/Donald and Barbara Zucker School of Medicine, Staten Island University Hospital, Staten Island, USA

**Keywords:** fibrous histiocytoma, skull invasion, scalp reconstruction, atypical fibroxanthoma

## Abstract

Malignant fibrous histiocytoma (MFH) is an undifferentiated high-grade pleomorphic sarcoma and is considered the most common primary soft tissue sarcoma in adults. MFH is known to arise in the trunk, extremities and retroperitoneum although it can arise anywhere in the body.MFH of the skin is uncommon and even less frequent is the involvement of the scalp, especially with skull invasion. Most of the MFH cases present as a gradually growing lesion over a period of one to two years and is associated with ulceration and hemorrhage in most of the cases. Treatment of MFH is usually surgical resection.

We present a case of MFH in an 85-year-old gentleman that was invading the skull which required a multidisciplinary surgical treatment for resection and microvascular free flap reconstruction.

## Introduction

Malignant fibrous histiocytoma (MFH) is a pleomorphic sarcoma, which is considered the most common primary soft tissue sarcoma in adults [1-5]. MFH is known to develop in the trunk, extremities and retroperitoneum although it can occur anywhere in the body [5]. MFH of the skin is uncommon especially in the scalp. We report a case of MFH of the scalp with invasion through the skull, which required a multidisciplinary surgical treatment for resection and microvascular free flap reconstruction.

## Case presentation

An 85-year-old gentleman with a history of basal cell carcinoma of the scalp seven years prior, which had been removed using Mohs chemosurgery, presented to his dermatologist with a complaint of a nodular indurated ulcerative lesion on the left parietal aspect of his scalp. The lesion had been present for several months, but the patient became concerned one month prior to presentation after his wife noticed bleeding and drainage of serous fluid from the lesion. The patient denied any other associated symptoms including headache, nausea, vision changes, numbness, tingling or weakness of either the upper or lower extremities.

An attempt was made to excise the lesion in the dermatologist’s office utilizing a Mohs procedure. However, the procedure was aborted due to concerns that the lesion might be invading the underlying skull. The patient was subsequently referred to a plastic surgeon and neurosurgeon for further evaluation and care.

**Figure 1:**
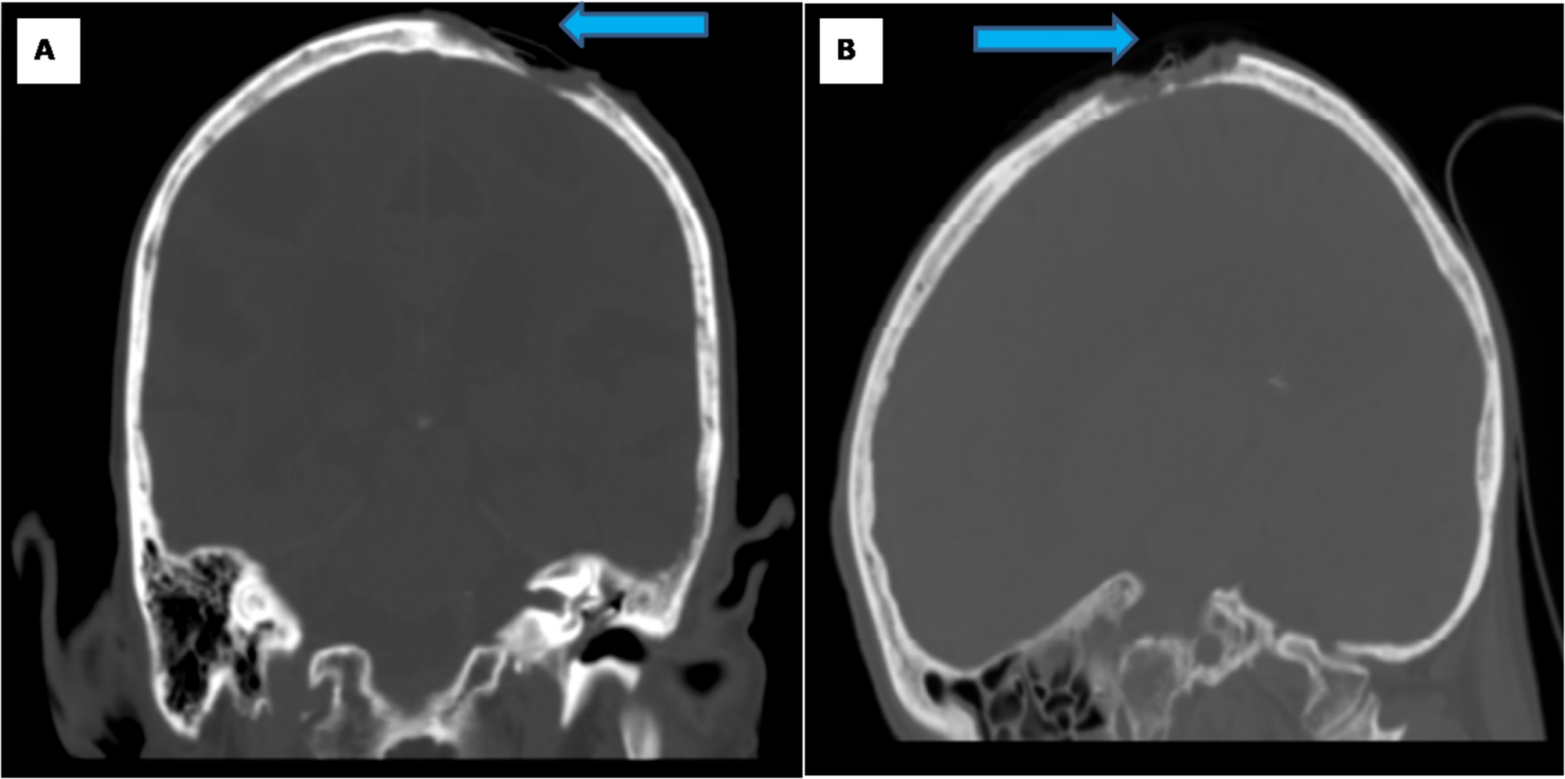
CT scan of the head There is a destructive lytic lesion involving the adjacent calvarium with severe thinning/destruction of the inner table (blue arrow). (A) Coronal view, (B) sagittal view.

Computerized tomography (CT) scan of the head (Figure 1) and magnetic resonance imaging (MRI) of the brain demonstrated erosion through both the outer and inner tables of the skull. There was no extension into the intracranial domain. Preliminary biopsy report from the lesion demonstrated atypical fibroxanthoma. After obtaining preoperative medical clearance, the patient was taken to the operating room for a combined procedure with neurosurgery and plastic surgery for en bloc resection and reconstruction. 

**Figure 2:**
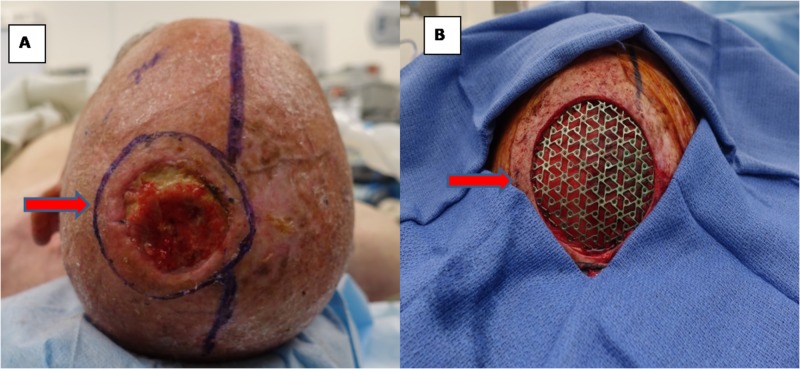
Intraoperative photographs of malignant fibrous histiocytoma (A) Scalp tumor with preoperative markings of the lesion with margins before resection (red arrow). (B) Scalp defect after duraplasty and cranioplasty with titanium mesh (red arrow).

**Figure 3:**
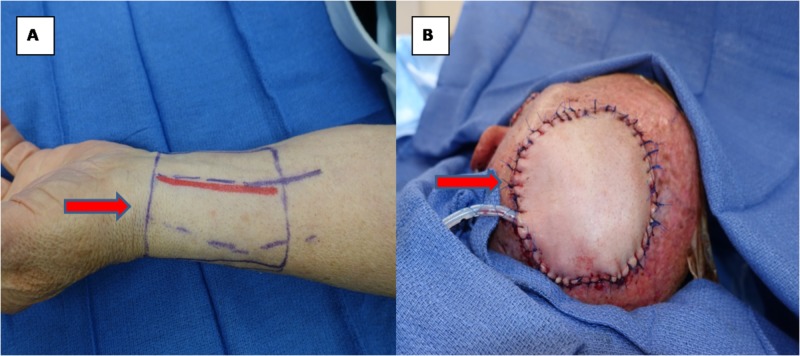
Intraoperative photographs of scalp defect reconstruction (A) Left radial forearm free flap donor site (red arrow). (B) Final scalp reconstruction using forearm free flap (red arrow).

The neurosurgery team performed a left craniectomy for an en bloc resection of the scalp and skull lesion followed by duraplasty and cranioplasty with titanium mesh. Subsequently, the plastic surgery team performed a microvascular free flap tissue reconstruction of the 7.5-cm-diameter circular scalp defect. A left radial forearm fasciocutaneous free flap was harvested and anastomosed to the superficial temporal vessels (Figures 2, 3: operative photos).

**Figure 4:**
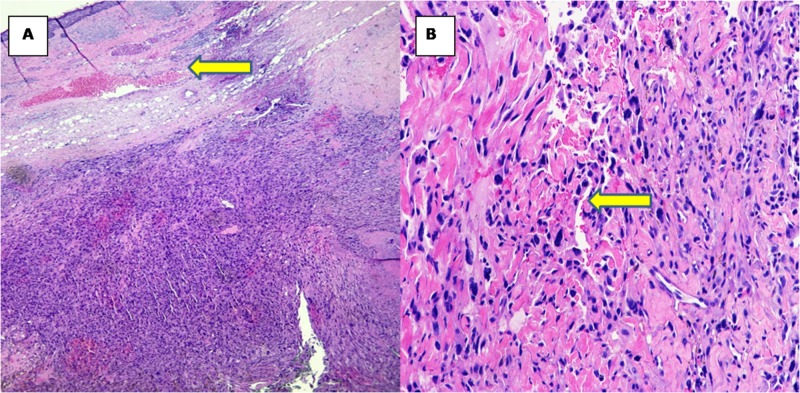
Histological findings of malignant fibrous histiocytoma All images were taken from glass slides stained with H&E (hematoxylin and eosin). (A) Low-power image 4X showing hypercellular spindle cell lesion in the dermis and subcutaneous tissue (yellow arrow). (B) 10X multiple pleomorphic nuclei are seen in the center of the image. Microscopically, malignant fibrous histiocytoma or atypical fibroxanthoma is composed of pleomorphic spindle cells (yellow arrow).

**Figure 5:**
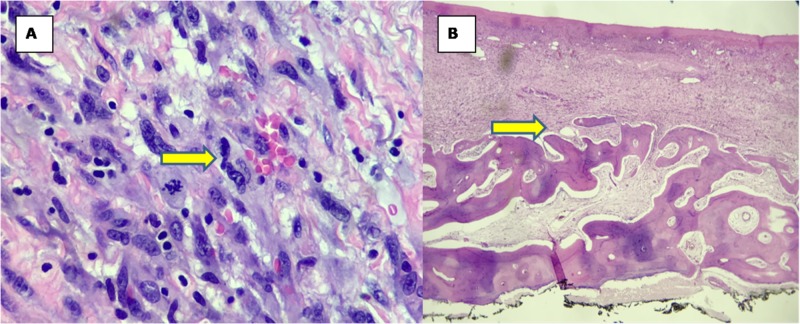
Histological findings of malignant fibrous histiocytoma (cont'd) All the images were taken from glass slides stained with H&E (hematoxylin and eosin). (A) 40X image taken from underneath the skin showing pleomorphic nuclei and mitosis and lymphocytic infltration (yellow arrow). (B) 4X image demonstrating involvement of the skull bone (yellow arrow).

The free flap was monitored clinically and with a handheld Doppler during the hospital stay. The patient was discharged home after an uncomplicated, eight-day hospital course. Review of the pathology specimen was consistent of the diagnosis of a MFH (atypical fibroxanthoma) with osseous invasion (Figures 4, 5: pathology slides).

## Discussion

MFH is an undifferentiated high-grade pleomorphic sarcoma and is considered the most common primary soft tissue sarcoma in adults [1,5]. MFH is known to arise in the trunk, extremities and retroperitoneum although it can arise anywhere in the body [2,5-9]. MFH of the skin is uncommon and even less frequent is the involvement of the scalp, especially with skull invasion [7]. Tumors of the scalp can be challenging to excise and usually required a complex reconstructive procedure [9]. MFH is rare as a primary cutaneous tumor because only a small proportion is usually located exclusively in the cutis [1,7]. It is diagnosed more frequently in men from middle age to late adult life and is very rarely diagnosed in children [5,7,8]. The incidence of malignant neoplasms of the scalp ranges from 0% to 7.7%, with a great majority that are considered to be metastatic rather than a primary tumor [5]. There is a paucity of literature regarding the prevalence of scalp and skull neoplasms. Review of the literature to date reported that MFH of the head and neck region is only 3% of all cases [10-12].

Most of the MFH cases present as a gradually growing lesion over a period of one to two years and is associated with ulceration and hemorrhage in most of the cases [5-7,10]. Pigmentation and erythema have also been reported [9]. If the tumor is located adjacent to bone, it may produce periosteal reaction or cortical erosion [7]. When located on the scalp, invasion can progress into the meninges, but is a rare occurrence [5-7]. Thus, radiological imaging of the head, whether CT scan or MRI, is indicated to evaluate the extent of the tumor. 

The differential diagnosis of MFH includes squamous cell carcinoma, malignant melanoma, keratoacanthoma, basal cell carcinoma, dermatofibrosarcoma, pleomorphic liposarcoma and leiomyosarcoma. Histological features and combined immunohistochemical markers are necessary for definitive diagnosis [1,5,7]. The dermatopathological and histological types of MFH include storiform-pleomorphic, myxoid, giant cell, inflammatory and angiomatoid tumors [1,7]. The storiform-pleomorphic is the most common MFH histologic type. Because of the histological similarity between MFH and atypical fibroxanthoma (AFX), AFX has been considered a superficial variant of MFH with lower malignant potential [1,7]. Size and depth aid in differentiation between AFX and MFH. AFX are characteristically nodular, tan to light-brown color lesions that are usually less than 2 cm. Half of AFX tumors are ulcerated [1]. Most of AFX (80%) are restricted to the reticular dermis, but may extend to the upper one third of the subcutaneous adipose tissue [1]. If a tumor extensively involves fascia, penetrates the muscle or displays necrosis, demonstrates perineural and vascular invasion, then it should be diagnosed as MFH. It has been hypothesized that locally invasive or metastasizing lesions may have initially been MFH, squamous cell carcinoma or malignant melanoma, rather than AFX [1,2].

Treatment of MFH is usually surgical resection with recommended 1-2 cm margin; however, the recurrence rate is up to 45% after "complete" surgical excision [1-4,6,7]. The reconstruction of partial- or full-thickness scalp defects encompasses many different techniques including the use of split-thickness skin grafts, full-thickness skin grafts, local or regional flaps, tissue expansion, Integra® (Integra Life Sciences, Plainsboro, NJ) and free tissue transfer [7,9]. Adjuvant radiotherapy and methotrexate-based chemotherapy have been shown to decrease the recurrence rate and improve survival; however, no firm conclusion can be drawn in regards to their effect as a treatment modality [5,7,11].

The overall rate of metastasis in MHF ranges from 5% to 55% with the most common sites for metastasis being the lung (90%), lymph nodes (35%), bone (8%) and the liver (1%) [1,5,7]. The tumor cells of MFH have the tendency to grow along fascial planes, thus making them more prone for local recurrence [5,11]. Tumor recurrences are commonly seen within the first two years [3,5,12]. Superficial lesions have been reported to recur more frequently than deep lesions [12].

## Conclusions

We reported a case of MFH of the scalp with invasion through the skull in an 85-year-old gentleman. CT scan of the head and MRI of the brain demonstrated erosion through both the outer and inner tables of the skull. Treatment of MFH required a multidisciplinary surgical treatment for resection and microvascular free flap reconstruction. Diagnosis of MFH is challenging, and it is important to differentiate MFH from other dermatologic pathologies, such as squamous cell carcinoma and malignant melanoma. Histological features and immunohistochemical markers are necessary for definitive diagnosis. Surgical excision is the definitive treatment. The excisional wound often does not permit primary closure and necessitates some form of complex reconstruction.
